# Persistence in the shadow of killers

**DOI:** 10.3389/fmicb.2014.00342

**Published:** 2014-07-14

**Authors:** Robert M. Sinclair

**Affiliations:** Mathematical Biology Unit, Okinawa Institute of Science and TechnologyOkinawa, Japan

**Keywords:** intra-species interaction, killer phenotype, competitive exclusion, coexistence, mathematical analysis

## Abstract

Killing is perhaps the most definite form of communication possible. Microbes such as yeasts and gut bacteria have been shown to exhibit killer phenotypes. The killer strains are able to kill other microbes occupying the same ecological niche, and do so with impunity. It would therefore be expected that, wherever a killer phenotype has arisen, all members of the population would soon be killers or dead. Surprisingly, (1) one can find both killer and sensitive strains in coexistence, both in the wild and in *in vitro* experiments, and (2) the absolute fitness cost of the killer phenotype often seems to be very small. We present an explicit model of such coexistence in a fragmented or discrete environment. A killer strain may kill all sensitive cells in one patch (one piece of rotting fruit, one cave or one human gut, for example), allowing sensitives to exist only in the absence of killer strains on the same patch. In our model, populations spread easily between patches, but in a stochastic manner: one can imagine spores borne by the wind over a field of untended apple trees, or enteric disease transmission in a region in which travel is effectively unrestricted. What we show is that coexistence is not only possible, but that it is possible even if the absolute fitness advantage of the sensitive strain over the killer strain is arbitrarily small. We do this by performing a specifically targeted mathematical analysis on our model, rather than via simulations. Our model does not assume large population densities, and may thus be useful in the context of understanding the ecology of extreme environments.

## Introduction

The Purpose of Computing Is Insight, Not Numbers (Hamming, 1987).

It will be useful to begin with a clear statement of what this work is really about. The central question being addressed is whether killer and sensitive phenotypes could in theory coexist in the same environment even if the absolute fitness penalty for the killer phenotype were arbitrarily small. The question is motivated by experimental and field observations to be described below, but is to be approached here from a theoretical point of view. This immediately implies that what is needed is a theoretical framework which has two apparently contradictory properties: First, it must capture important aspects of the relevant biology. Second, it must be simple enough that the question can be answered. This theoretical framework will therefore necessarily be a compromise between realism and tractability. Furthermore, what is actually needed is only a single affirmative result, since that proves the theoretical possibility of coexistence. The phrase “arbitrarily small” excludes some standard approaches, and it is best to make this point now rather than later, because it is this compact phrase which drags us into what will be unfamiliar territory for many readers. If the theoretical framework were to be in the form of a standard numerical simulation of a model, then the best one could do would be to show that coexistence is possible for given small absolute fitness penalties for the killer phenotype. This is not the same as “arbitrarily small”: if a simulation were to show coexistence to be possible for a fitness penalty of 0.1, it would still leave open the question of whether coexistence could be possible for a fitness penalty of 0.01 and so on ad infinitum. We are thus motivated, by the question we have chosen to investigate, to search outside of the box of standard scientific computing tools until a truly suitable approach is found. The field of mathematical analysis (Ross, 1980) offers itself at this point, since it includes powerful techniques for dealing with the arbitrarily small. Moreover, we can apply elementary methods of mathematical analysis to mathematical models. We are now ready to sketch our approach: we will analyse a model which captures much of the biology but is simple enough to allow a mathematical analysis to be performed. If we can show that coexistence of killer and sensitive phenotypes is possible, for arbitrarily small absolute fitness penalties for the killer phenotype, for this single model, then we will have answered our question in the affirmative. Once this has been done, the particular model is no longer of direct importance (in the same sense that a certain telescope may be used to make an important astronomical observation, but it is almost always the observation which has lasting importance, not the telescope), although we hope it may be useful in other investigations. To paraphrase Hamming, the purpose of our analysis is insight, not the establishment of a computational model *per se*. In other words, the reader should not be expecting to see the standard modeling approach of computational biology, with all of the standard parameter fitting and graph plotting that entails. Instead, the reader should expect to find here something rather unusual, a more truly mathematical approach, but something which has already been proposed in a related context (Silva, 2011).

The phenomenon of killer phenotypes, which possess the ability to kill conspecifics while being themselves immune (Marquina et al., 2002; Breinig et al., 2006), is widespread in the microbial world (Schmitt and Breinig, 2006; Schrallhammer, 2010; Holt et al., 2013). As more such systems are studied, it is becoming increasingly clear that the evolutionary contexts are so varied (Cornejo et al., 2009 provide a surprising example) that it may be impossible to encompass all that is relevant (such as biodiversity Czárán et al., 2002) in a single, simple model. Classical theory predicts that competition for a single resource should result in the survival of only one competitor (Hardin, 1960), and yet sensitive strains can be more common than killers (Riley and Gordon, 1999; Pieczynska et al., 2013), and it has been observed that there can be coexistence between killer and sensitive strains, necessitating the development of new models (Czárán and Hoekstra, 2003; Vadasz et al., 2003). Here, we provide a novel explicitly solvable mathematical model of the population dynamics of a species with killer and sensitive strains inhabiting a fragmented but potentially highly interconnected environment. Our model includes only killer and sensitive phenotypes. While we were originally inspired by the image of fallen, over-ripe fruits beneath a grove of fruit trees, with spores providing the mechanism of connectivity, the mathematical structure of the model allows it to be applied to many other situations: enteric pathogens live in isolated environments (within the digestive tracts of their individual hosts), but transmission between hosts does occur and can represent a high degree of connectivity in the case of a pandemic. Also, intraterrestrial microbial communities living in largely isolated caves or niches may be sporadically connected by flooding events (Hawes, 1939), as could psychrophilic microbial communities living in niches in or on ice (Margesin and Miteva, 2011) be connected during annual thawing or via other dispersal mechanisms.

Rather surprisingly, it has been shown that the cost of toxin production can be negligible (Garbeva et al., 2011), and is presumably only a few percent when measurable (Wloch-Salamon et al., 2008). We asked whether, in a model, coexistence of killer and sensitive phenotypes is possible for any difference in absolute fitness between killer and sensitive phenotypes, however small. That requires analysis rather than simulation, and this point has therefore decisively influenced our approach.

## Materials and methods

We describe here an explicitly solvable model of yeast population dynamics on an infinite number of patches, in which killer and sensitive strains can coexist. Our model includes killer and sensitive strains only. In the following, we will use the example of a killer yeast in our verbal descriptions of the model. A full mathematical treatment would not be appropriate here. We will instead provide what may be called a sketch of the model and our analysis of it. The Supplementary Material contains details of the most important part of the mathematical analysis, but it is also best described as a sketch rather than a formal proof.

Each patch is intended to represent a single piece of fruit. A patch can be colonized by spores from any patch. If a patch is colonized only by spores of the sensitive yeast strain, then the patch will emit only spores of the sensitive strain. If a killer yeast spore lands on a patch, then any sensitive yeast colony will be eradicated, and the patch will emit only spores of the killer yeast. If a sensitive yeast spore lands on a patch colonized by killer yeast, it will not survive nor influence the (killer yeast) spore production of the patch. The number of spores emitted by a patch depends only upon the type of yeast that has successfully colonized it. If no spores have landed on a patch then that patch will emit no spores. Sporulation occurs in all patches simultaneously, leaving all patches barren and ready for the next cycle, initialized by the dispersal of the spores.

Let *f_S_* ≥ 1 and *f_K_* ≥ 1 denote the average number of spores emitted per patch colonized by sensitive (S) or killer (K) strains, where these are to be understood as effective rather than absolute values, since the model assumes that all spores are viable and eventually find a patch. As expected, these numbers play the role of absolute fitnesses. Also, let 0 ≤ *x_S_* ≤ 1 and 0 ≤ *x_K_* ≤ 1 denote the respective fractions of patches successfully colonized (at the time of sporulation) by the two strains.

The dynamics of the killer yeast strain is not in any way influenced or restricted by the sensitive strain, and so can be treated independently. The probability of a given patch not being reached by any killer strain spore is

$$e^{-f_{K^{x_{K}}}}.$$

The reason for this can be understood by first considering a finite number of patches, and then taking the limit as that number goes to infinity. Let *n* denote the (finite) number of patches. The probability that any given spore will not land on any given patch is 1−1/*n*, assuming random dispersal. The total number of colonized patches is *n x_K_*, so the average number of spores emitted in total is *n f_K_ x_K_*. The probability that none of these land on any given patch is (1−1/*n*)^*n f_K_ x_K_*^. If we now let *n* go to infinity, we find that we can quite directly use one of the standard definitions of the exponential function:

$$\begin{array}{l} \underset{n\;\to \;\infty }{\lim}{\left(1-\frac{1}{n}\right)}^{nf_{K}x_{K}}=\underset{n\;\to \;\infty }{\lim}{\left[{\left(1-\frac{1}{n}\right)}^{n}\right]}^{f_{K}x_{K}}\\ \;={\left[e^{-1}\right]}^{f_{K}x_{K}}=e^{-f_{K}x_{K}}. \end{array}$$

This will be, for an infinite number of patches, the fraction of patches which are not reached by any spore. On the other hand, the fraction of patches which are reached by a spore must be the remainder, or 1 − *e^−f_K_x_K_^*. The killer strain population dynamics is therefore described by the map

(1) $$\begin{array}{cc} x_{K}\mapsto \left(1-e^{-f_{K}x_{K}}\right). & \left(1\right) \end{array}$$

Since we can write

$$x_{K}\mapsto f_{K}x_{K}+O\left(f_{K}^{2}x_{K}^{2}\right),$$

we can state that *f_K_* plays the role of absolute fitness for small *f_K_ x_K_*. In other words, our model includes the phenomenon of exponential growth when resources are not a limiting factor, and this exponential growth can be used to define an absolute fitness.

According to standard theory, the map (Equation 1) has an unstable fixed point at *x_K_* = 0 and a stable fixed point at

$$x_{K}=X_{K}=1+\frac{W\left(-f_{K}e^{-f_{K}}\right)}{f_{K}},$$

where *W* is the principal branch of the Lambert W function (Corless et al., 1996).

The dynamics of the sensitive strain is governed by the same equations in the complete absence of spores of the killer strain. The reason for this assumption is the observation (discussed above) that the difference in absolute fitness between the killer and sensitive strains can be very small. In the presence of an established killer strain population occupying a fixed fraction (*X_K_*) of all patches, the sensitive strain population dynamics is determined by the map

$$x_{S}\mapsto \left(1-e^{-f_{S}x_{S}}\right)\left(1-X_{K}\right),$$

which has an unstable fixed point at *x_S_* = 0 and a stable fixed point at

$$x_{S}=X_{S}=\left(1-X_{K}\right)+\frac{W\left(-\left(1-X_{K}\right)f_{S}e^{-\left(1\;-\;X_{K}\right)f_{S}}\right)}{f_{S}}$$

if and only if

(2) $$\begin{array}{cc} f_{S}>\frac{-f_{K}}{W\left(-f_{K}e^{-f_{K}}\right)}. & \left(2\right) \end{array}$$

**Figure 1:**
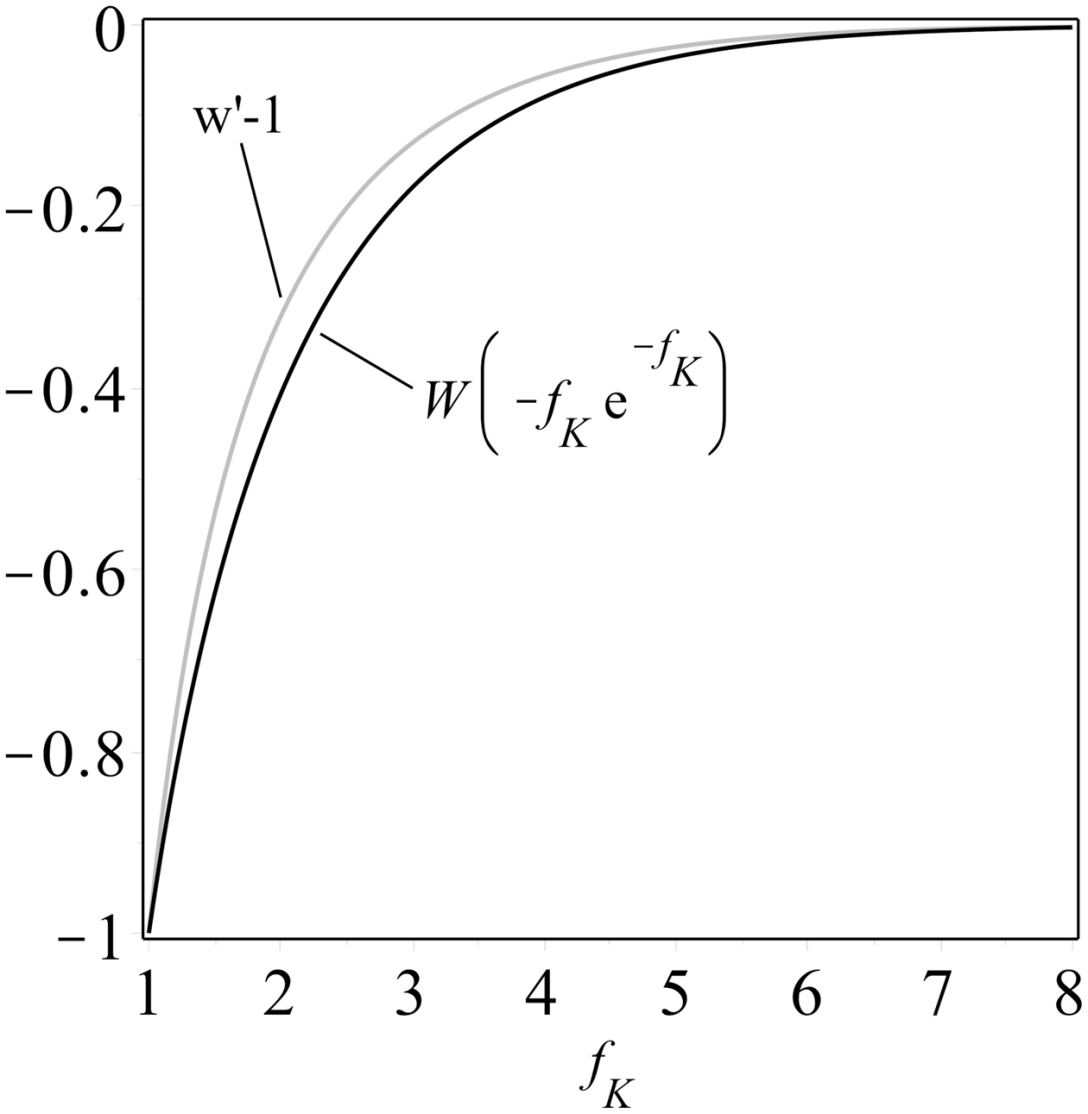
**A comparison of the composition of an exponential function and the Lambert W function, *W (−f_K_ e^−f_K_^*), and the upper bound derived in the Supplementary Material, both plotted as functions of *f_K_* ≥ 1.** Note that both curves are monotonically increasing, a property which facilitates analysis.

One can construct (details are in the Supplementary Material and see also Figure 1) the upper bounds

(3) $$\begin{array}{cc} 5f_{K}-4>\frac{f_{K}^{2}}{3-2f_{K}}\geq \frac{-f_{K}}{W\left(-f_{K}e^{-f_{K}}\right)} & \left(3\right) \end{array}$$

for 1 < *f_K_* < 1.09. If the cost of the killer phenotype is δ > 0, so that *f_K_* = *f_S_* − δ, and we set *f_S_* = 5*f_K_* − 4, then for *any* very small choice of 0 < δ < 0.36, we can state that coexistence is possible in our model, and can provide an explicit *family* of examples, with *f_K_* = 1 + δ/4 and *f_S_* = 1+5δ/4.

Given a stable subpopulation of the killer strain, a necessary condition for establishment of a subpopulation of the sensitive strain from a finite number of sensitive spores is

$$f_{S}\left(1-X_{K}\right)>1,$$

which is identical to the previous inequality (Equation 2). We omit the technical details here, but the product *f_S_* (1 − *X_K_*) is the effective absolute fitness of the sensitive strain in the presence of an established population of the killer strain. For each patch successfully colonized by a sensitive strain, an average of *f_S_* spores will be emitted, but only 1 − *X_K_* of patches are free of the killer strain, so only this fraction is will survive to sporulation. These considerations apply equally to an initial exponential growth phase or a stable state, and therefore the agreement with Equation (2) is to be expected.

The total fraction of patches stably colonized by either strain is

$$X_{K}+X_{S}=1+\frac{W\left(-\left(1-X_{K}\right)f_{S}e^{-\left(1-X_{K}\right)f_{S}}\right)}{f_{S}}<1.$$

Note that

$$X_{K}+X_{S}>X_{K}$$

if Equation (2) is satisfied, meaning that the sensitive strain, when present, only contributes to total population.

Can any ratio of killer to sensitive phenotypes be achieved in this model? Furthermore, can any total fraction of patches be stably colonized? Since *X_K_* and *X_S_* are continuous functions of *f_K_* and *f_S_*, and

$$\begin{array}{l} \underset{f_{K}\;\to \;\infty }{\lim}X_{K}=1,\\ \underset{f_{S}\;\to \;\infty }{\lim}X_{S}=1-X_{K},\\ \underset{f_{K}\;\to \;0}{\lim}X_{K}=\underset{f_{S}\to -f_{K}/W\left(-f_{K}e^{-f_{K}}\right)}{\lim}X_{S}=0 \end{array}$$

and

$$\underset{f_{K}\;\to \;\infty }{\lim}\left(X_{K}+X_{S}\right)=\underset{f_{S}\;\to \;\infty }{\lim}\left(X_{K}+X_{S}\right)=1,$$

all pairs (*X_K_, X_S_*) for which *X_K_* + *X_S_* < 1 holds can be achieved by suitable choices of *f_K_* or *f_S_*.

As a numerical example, if *f_K_* = 2 and *f_S_* = 5, then Equation (2) is satisfied, and two subpopulations of sizes *X_K_* ≈ 0.797 (i.e., 79.7% of patches) and *X*_S_ ≈ 0.006 (i.e., 0.6% of patches) can stably coexist. If one were interested in trying to fit a minimalist model of this type to real data, note that it would not be enough to know the ratio of killer-dominated patches to sensitive-colonized patches. One also needs the fraction of patches which are colonized by neither strain, data which is not always reported in the literature.

## Results

Two direct consequences of the model are (1) that killer and sensitive strains can coexist in any given proportion, and (2) that the presence of a sensitive subpopulation increases the total population size of yeast (including both strains) without reducing the population size of the virus population maintained by the killer yeast strain. Taking a broader point of view, the second consequence means that the species benefits from having both sensitive and killer strains.

### Coexistence for arbitrarily small cost of killer phenotype

Since our model is explicitly solvable, we are able to perform a mathematical analysis which showed (see the Equation 3 and the associated comments above) that coexistence is possible for any extra fitness cost of the killer phenotype, *however small*.

As a numerical example, if *f_S_* = 1.0001, then we have coexistence for *f_K_* = 1.000049, and the very small fitness cost represented by δ = 0.000051. The corresponding fractions are *X_K_* ≈ 0.0000996 and *X_S_* ≈ 0.00000399. One notices that very small differences in fitness are achieved by populations for which the total fraction of colonized patches is also very small. This is the reason to suggest that this model may best be suited to extreme environments.

Using the explicit formulae from our analysis, *f_K_* = 1 + δ/4 and *f_S_* = 1 + 5δ/4, for a target cost of δ = 0.00004, we have *f_K_* = 1.00001 and *f_S_* = 1.00005, with the respective fractions being *X_K_* ≈ 0.00002 and *X_S_* ≈ 0.00006. Here we see the power of the analysis: we are able to construct infinitely many further such examples for even smaller values of δ, without lower limit.

Therefore, we are able to construct pairs (*f_K_, f_S_*) for which coexistence is guaranteed, and, furthermore, do so for any given fitness cost δ for the killer phenotype, *however small*.

## Discussion

It is not intuitively obvious that sensitive strains can survive in the presence of killers, given that our model has no fixed barriers to prevent the sensitive strains from being eradicated by encounters with killers. The value of our model lies not only in this prediction, which is consistent with other, related, models (the semi-analytical configuration-field approximations for the one- and two-species SCA models of Czárán and Hoekstra, 2003 in particular), but also in the fact that it is *explicitly solvable*, a property which allows types of analysis to be performed which are truly complementary to what is possible with simulations alone (Silva, 2011).

We have been able to prove that killer-sensitive coexistence is possible for any fitness penalty of the killer phenotype, *however small*. This is important because it has been shown that there does not have to be any measurable fitness cost for antibiotic production (Garbeva et al., 2011). The fact that our model applies naturally to communities with low total population densities suggests that it may be applicable to microbial communities in extreme environments.

### Conflict of interest statement

The author declares that the research was conducted in the absence of any commercial or financial relationships that could be construed as a potential conflict of interest.
