# Supplementary material for: Persistence in the shadow of killers
Source: Front Microbiol. 2014 Jul 14;5:342. doi: 10.3389/fmicb.2014.00342 (PMC4095038; doi:10.3389/fmicb.2014.00342)
Supplement: Supplementary file 1 [file Presentation1.PDF]

Persistence in the Shadow of Killers

Robert Sinclair[1]*

[1]Mathematical Biology Unit, OIST, Okinawa, Japan

*** Correspondence:** Robert Sinclair, Mathematical Biology Unit, Okinawa Institute of Science and Technology Graduate University, 1919-1 Tancha, Onna-son, Okinawa, 904-0495, Japan.
sinclair@oist.jp

Appendix

Here, we will sketch a derivation of one of the upper bounds (the more difficult one) of Equation (3). Let us write it as

$$\frac{f_K^2}{3-2f_K} \geq \frac{-f_K}{W(-f_K e^{-f_K})}\,.$$

The difficulty lies in the fact that the Lambert W function is not defined explicitly, but in terms of the solution to an equation: $W(z)$ is the quantity which satisfies

$$z = W(z)\, e^{W(z)}\,.$$

Our strategy will be to substitute this equation for a simpler one, which we will be able to solve explicitly. What follows are technical details.

In our case, in the place of z we have $-f_K e^{-f_K}$, a quantity which varies between $-e^{-1}$ and 0 (recall that $f_K \geq 1$). When $f_K = 1$ we have $z = -f_K e^{-f_K} = -e^{-1} = -1 \times e^{-1}$, which allows us to deduce the (well-known) special value

$$W(-e^{-1}) = -1\,.$$

This provides us with a very useful point of reference. Let us capture the fact by writing

$$W(-f_K\, e^{-f_K}) = -1 + w = w - 1\,,$$

where w literally means nothing more than how far $W(-f_K\, e^{-f_K})$ deviates from minus one. It assumes values between zero and one. Now we can say that w is the quantity which satisfies

$$z = (w-1)\, e^{w-1}\,.$$

Of course we know that $w = W(-f_K\, e^{-f_K}) + 1$, but W itself remains mysterious.

How can we get around this problem? There is a way, and our starting point will be the standard bounds on the exponential function

$$1 + x \le e^x \le \frac{1}{1-x}$$

which are simultaneously true for $x < 1$. It turns out that direct use of these inequalities does not provide us with a bound which is strong enough for our purposes, so we first make use of the fact that $e^x = e^{x/2}\, e^{x/2}$ to write

$$e^x \;=\; e^{\frac{x}{2}}\, e^{\frac{x}{2}} \;\le\; \frac{1}{1-\frac{x}{2}}\,\frac{1}{1-\frac{x}{2}} \;=\; \frac{1}{\frac{1}{4}x^2 - x + 1},$$

for $x < 2$, and

$$e^{x-1} = \frac{e^x}{e} \le \frac{1}{e\left(\frac{1}{4}x^2 - x + 1\right)},$$

from which it follows that

$$(w-1)\, e^{w-1} \;\ge\; \frac{w-1}{e\left(\frac{1}{4}w^2 - w + 1\right)},$$

where we have replaced x with $W(-f_K\, e^{-f_K}) = w - 1$ and also multiplied both sides by $w - 1$, which is negative for $f_K \ge 1$. Putting this together with the definition of W, we have

$$-f_K\, e^{-f_K} \;=\; W(-f_K e^{-f_K})\, e^{W(-f_K e^{-f_K})} \;=\; (w-1)\, e^{w-1} \;\ge\; \frac{w-1}{e\left(\frac{1}{4}w^2 - w + 1\right)}.$$

Solving

$$-f_K\, e^{-f_K} = \frac{w'-1}{e\left(\frac{1}{4}[w']^2 - w' + 1\right)}$$

for w' will give us an upper bound on $w = W(-f_K\, e^{-f_K}) + 1$. The solution is

$$w' = \frac{2\left(f_K\, e^{1-f_K} + \sqrt{1 - f_K\, e^{1-f_K}} - 1\right)}{f_K\, e^{1-f_K}}.$$

finally providing us with an upper bound on $W(-f_K\, e^{-f_K})$ in terms of well-known elementary functions. This bound is

$$W(-f_K\, e^{-f_K}) \;\le\; \frac{2\left(f_K\, e^{1-f_K} + \sqrt{1 - f_K\, e^{1-f_K}} - 1\right)}{f_K\, e^{1-f_K}} - 1\,.$$

Figure 1 illustrates this bound, comparing the actual curve of $W(-f_K\, e^{-f_K})$ and the curve of the

upper bound $w' - 1$, both as functions of f_K.

The next step is to apply this bound to Equation (3). We obtain

$$\frac{-f_K}{W(-f_K\, e^{-f_K})} \leq \frac{-f_K^2\, e^{1-f_K}}{f_K\, e^{1-f_K} + 2\sqrt{1 - f_K\, e^{1-f_K}} - 2}$$

but this is still rather complicated, so we proceed to simplify while maintaining the property of being an upper bound. Since $f_K \geq 1$, we have $0 < e^{1-f_K} \leq 1$, so we can immediately write

$$\frac{-f_K}{W(-f_K\, e^{-f_K})} \leq \frac{-f_K^2}{f_K\, e^{1-f_K} + 2\sqrt{1 - f_K\, e^{1-f_K}} - 2} = \frac{f_K^2}{(2 - f_K\, e^{1-f_K}) - 2\sqrt{1 - f_K\, e^{1-f_K}}}.$$

The function $2 - f_K\, e^{1-f_K}$ has the first derivative $e^{1-f_K}\,(f_K - 1)$, which is non-negative for all $f_K \geq 1$. It easily follows that

$$1 \leq 2 - f_K\, e^{1-f_K} \leq 2\,,$$

so we can continue to simplify our upper bound. Now we have

$$\frac{-f_K}{W(-f_K\, e^{-f_K})} \leq \frac{f_K^2}{1 - 2\sqrt{1 - f_K\, e^{1-f_K}}}, \tag{4}$$

at least as long as $1 - 2\sqrt{1 - f_K\, e^{1-f_K}} \neq 0$. From the standard lower bound $e^x \geq 1 + x$, which is true for all x, we know that $e^{1-f_K} \geq 2 - f_K$ for all possible values of $f_K \geq 1$. We have

$$1 - 2\sqrt{1 - f_K\, e^{1-f_K}} \geq 1 - 2\sqrt{1 - f_K\,(2 - f_K)} > 0$$

but also

$$1 - 2\sqrt{1 - f_K\,(2 - f_K)} = 1 - 2\sqrt{f_K^2 - 2f_K + 1} = 1 - 2\sqrt{(f_K - 1)^2} = 3 - 2\, f_K$$

for $f_K \geq 1$, so we actually demand

$$1 - 2\sqrt{1 - f_K\, e^{1-f_K}} \geq 3 - 2\, f_K > 0\,,$$

which is satisfied if $1 \leq f_K < 3/2$.

Returning to Equation (4), if we make use of the standard bound $e^x \geq 1 + x$ once again, but with $x = 1 - f_K$, we arrive (again – making use of calculations already performed above) at

$$\sqrt{1 - f_K\, e^{1-f_K}} \leq \sqrt{1 - f_K\,(2 - f_K)} = \sqrt{(f_K - 1)^2} = f_K - 1\,,$$

which finally brings us to the form in Equation (3), still assuming $1 \leq f_K < 3/2$:

$$\frac{-f_K}{W(-f_K\, e^{-f_K})} \leq \frac{f_K^2}{3 - 2\, f_K}\,.$$
